# Fc-engineering for improved cancer immunotherapy

**DOI:** 10.1042/BST20250554

**Published:** 2026-08-20

**Authors:** Ella Borgman, Camille Le Gall, Hidde Ploegh, Novalia Pishesha

**Affiliations:** 1 Harvard College, Cambridge, Massachusetts, U.S.A.; 2 Division of Immunology, Boston Children’s Hospital, Boston, Massachusetts, U.S.A.; 3 Department of Pediatrics, Harvard Medical School, Boston, Massachusetts, U.S.A.; 4 Program in Cellular and Molecular Medicine, Boston Children’s Hospital, Boston, Massachusetts, U.S.A.

**Keywords:** antibodies, cancer, protein engineering

## Abstract

Monoclonal antibodies have revolutionized cancer therapy, with approved therapeutics belonging predominantly to the immunoglobulin G (IgG) class. These IgGs mediate antitumor effects predominantly through engagement of Fcγ receptors on immune cells. This engagement results in therapy-activating Fc effector functions such as antibody-dependent cellular cytotoxicity, antibody-dependent cellular phagocytosis, and complement-dependent cytotoxicity. Limitations nonetheless include suboptimal effector engagement and adverse immunological side effects. These drawbacks have driven the search for and optimization of Fc-engineering strategies, including Fc mutations, glycan modification, and choice of Ig isotype, to enhance therapeutic efficacy and tailor immune interactions.

## Introduction

In 1997, the first cancer monoclonal antibody (mAb), rituximab, was approved. It targets the CD20 B-cell antigen and has proven extremely effective in treating B-cell lymphomas [1]. Since the approval of rituximab, mAbs have reshaped the cancer treatment landscape and ignited the concept of targeted cancer immunotherapy. Today, there are 66 FDA-approved antitumor antibody treatments [2].

Nearly all the approved full-length mAbs for cancer are of the immunoglobulin (IgG) isotype, most commonly IgG1, with IgG2 and IgG4 also represented depending on the target and the desired Fc function. MAbs used to treat other diseases are also predominantly IgG. Nebacumab, a human IgM mAB developed for the treatment of sepsis, was withdrawn in 1993 after failing to improve survival rates in clinical trials [3]. A significant advantage of IgG compared with other isotypes (IgM, IgA, IgE, and IgD) is its extended half-life due to its unique interaction with the neonatal Fc receptor (FcRn). This interaction helps protect IgG from degradation [4]. IgA and IgM typically have half-lives of 5–7 days, while natural IgG can have half-lives of more than 3 weeks. The rescue of IgG from degradation is a pH-dependent recycling process [4]. FcRn binding to the Fc region of IgG does not occur at physiological pH (7.4), but does occur at an acidic pH of 6 or below. When IgG is in an endosomal compartment, FcRn retains the IgG and allows its recycling to the cell surface, where it is released, thus avoiding transport to lysosomes for degradation. A longer plasma half-life provides the benefit of lower and less frequent dosing in patients and increases the likelihood that the treatment will reach its target.

In addition to FcRn, there are numerous IgG Fc-engaging effector molecules that increase IgG’s ability to clear and kill target cells, including cancer cells. IgG interacts with Fc gamma receptors (FcγR) expressed by myeloid cells, natural killer cells, and some lymphoid cells, where the Fc receptors initiate a range of effector functions [5]. FcγR engagement links antigen recognition by an antibody to several effector mechanisms; however, one receptor does not always map to one function. FcγRIIIa (CD16A) engagement on NK cells is a major route to antibody-dependent cellular cytotoxicity (ADCC), but FcγRIIIa is also expressed by monocytes and macrophages, where it can contribute to antibody-dependent cellular phagocytosis (ADCP). FcγRI and FcγRIIa are important activating receptors for macrophage- and neutrophil-mediated phagocytosis. Indeed, for several tumor-targeting antibodies, including anti-CD20 antibodies, monocyte/macrophage-mediated ADCP, rather than NK-cell ADCC, can be a principal mechanism of target-cell depletion, with the relative contribution of each pathway depending on antibody isotype, FcγR expression, and polymorphism, target-antigen density, and tissue context [6–9]. When bound to antigen, the IgG Fc portion can also bind to complement (C1q) and trigger complement-dependent cytotoxicity (CDC).

Fc-driven cytokine release is a further consequence of FcγR engagement that is important for cancer therapeutic antibodies. Beyond cytotoxicity and phagocytosis, FcγR cross-linking can induce cytokine release from myeloid and other FcγR-expressing cells. This can support productive antitumor immunity through immune-cell activation and antigen presentation, but excessive Fc- or immune-complex-driven activation can also cause cytokine release syndrome (CRS). TGN1412, a CD28 superagonist designed to treat leukemia, became infamous in its Phase 1 clinical trial for all six healthy volunteers suffering from cytokine storms that resulted in multiple organ failures soon after injection. This illustrates that antibody subclass selection alone does not guarantee Fc inertness, because CD32 (FcγRII) engagement contributed to the cytokine response despite an IgG4 format [10,11]. For T-cell-engaging bispecific antibodies, CRS is triggered by target-dependent T-cell activation and amplified by the myeloid compartment [12–14]. Accordingly, Fc-engineered antibodies should be evaluated not only for ADCC, ADCP, and CDC, but also for cytokine release, immune-complex behavior, and FcγR-crosslinking potential.

The other antibody isotypes maintain some of the Fc effector functions of IgG and can also access distinct Fc effector activities through their unique Fc regions (Table 1). IgM is a pentamer, providing multiple binding sites for C1q, which makes this isotype particularly effective in activating the complement cascade. Although IgM lacks the FcγR interactions described for IgG, another Fc-receptor, FcμR, interacts with IgM and contributes to B cell development, maturation, activation, humoral immunity, and immune tolerance [15].

**Table 1:** Summary of human and mouse Fc receptors (FcRs). Adapted from FcR literature review [85]

Receptor (common/CD name	Main Ig ligand(s)	Dominant function	Cell type expression
**Human FcRs**			
**FcγRI (CD64)**	IgG (IgG1, IgG3; IgG4) *high-affinity	**Activation**	Monocyte/macrophage and dendritic cell **Induced expression:** neutrophil and mast cell
**FcγRIIA (CD32A)**	IgG (all subclasses)	**Activation**	Monocyte/macrophage, neutrophil, dendritic cell, basophil, mast cell, eosinophil, and platelet
**FcγRIIB (CD32B)**	IgG (all subclasses)	**Inhibition**	B cell, dendritic cell, and basophil **Expression on subpopulation:** monocyte/macrophage and neutrophil
**FcγRIIC (CD32C)**	IgG (all subclasses)	**Activation**	Natural killer cell, monocyte/macrophage, and neutrophil
**FcγRIIIA (CD16A)**	IgG (all subclasses) *high-affinity for IgG3	**Activation**	Natural killer cell, monocyte/macrophage
**FcγRIIIB (CD16B)**	IgG (IgG1, IgG3)	**Activation/regulatory**	Neutrophil **Expression on subpopulation:** basophil
**FcRn**	IgG (all subclasses) *high-affinity	**IgG recycling/transport**	Monocyte/macrophage, neutrophil, dendritic cell, endothelium, epithelium, syncytiotrophoblast (outer layer of placenta)
**TRIM21**	IgG (all subclasses)	**Activation/proteasome viral degradation**	B cell, T cell, natural killer cell, monocyte/macrophage, neutrophil, dendritic cell, basophil, mast cell, eosinophil **Expression on subpopulation:** endothelium
**FcRL5 (CD307e)**	IgG (all subclasses) *variable for IgG2	**Activation**	B cell
**FcεRI**	IgE *high-affinity	**Activation**	Mast cell, basophil, platelet **Expression on subpopulation:** monocyte/macrophage, dendritic cell, eosinophil
**FcεRII (CD23)**	IgE	**Regulatory**	B cell, eosinophil, platelet **Induced expression:** monocyte/macrophage, dendritic cell **Expression on subpopulation:** epithelium
**FcαRI (CD89)**	IgA	**Activation**	Monocyte, neutrophil, eosinophil **Expression on subpopulation:** macrophage, dendritic cell
**FcRL4 (CD307d)**	IgA, possibly IgG3 and IgG4	**Inhibition/activation**	**Expression on subpopulation:** B cell
**Fcα/μR (CD351)**	IgM, IgA	**Phagocytosis/endocytosis**	Follicular dendritic cell **Expression on subpopulation:** B cell
**FcμR**	IgM	**Regulatory/ endocytosis**	B cell **Expression on subpopulation:** T cell, natural killer cell
**PolyIgR**	IgM, IgA	**Ig transport**	Epithelium

Receptor	Main Ig ligand(s)	Dominant function	Cell types expressed on
**Mouse FcRs**			
**FcγRI**	IgG (IgG2a, IgG2b, possibly IgG3) *high-affinity for IgG2a	**Activation**	**Induced expression:** monocyte/macrophage, dendritic cell
**FcγRIIB**	IgG (IgG1, IgG2a, IgG2b), IgE	**Inhibition**	B cell, monocyte/macrophage, neutrophil, dendritic cell, basophil, mast cell, eosinophil
**FcγRIII**	IgG (IgG1, IgG2a, IgG2b), IgE	**Activation**	Natural killer cell, monocyte/macrophage, neutrophil, dendritic cell, basophil, mast cell, eosinophil
**FcγRIV**	IgG (IgG2a, IgG2b), IgE *high-affinity for IgG2a, IgG2b	**Activation**	Monocyte/macrophage, neutrophil
**FcRn**	IgG (IgG1, IgG2a, IgG2b, IgG3), *high-affinity for IgG1	**IgG recycling/transport**	Monocyte/macrophage, neutrophil, dendritic cell, endothelium, yolk sac **Expression on subpopulation:** epithelium
**TRIM21**	IgM, IgG (IgG1, IgG2a, IgG2b)	**Activation/ proteasome viral degradation**	B cell, T cell, natural killer cell, monocyte/macrophage, neutrophil, dendritic cell, basophil, mast cell, eosinophil **Expression on subpopulation:** endothelium
**FcεRI**	IgE *high-affinity	**Activation**	Basophil, mast cell
**FcεRII**	IgE	**Regulatory**	B cell **Expression on subpopulation:** dendritic cell
**Fcα/μR**	IgM, IgA	**Phagocytosis/endocytosis**	B cell, follicular dendritic cell
**FcμR**	IgM	**Activation/endocytosis/regulatory**	B cell
**PolyIgR**	IgM, IgA	**Ig transport**	epithelium

The IgA isotype is associated with mucosal immunity and specifically engages the Fc receptor FcαRI (also known as CD89), expressed on neutrophils and macrophages, to trigger phagocytosis, degranulation, and cytokine release [16]. IgE, implicated in allergic responses and the defense against parasites, engages the FcεRI receptor found on mast cells and basophils [16]. This engagement leads to degranulation and the release of inflammatory mediators.

Given the robust ability of natural IgG to mediate effector functions and its long plasma half-life, IgG has remained the preferred isotype for therapeutic applications. Considerable effort has gone into engineering the Fc region of IgG to enhance the efficacy and durability of IgG-based treatments while minimizing adverse side effects. Recently, there has been growing interest in exploiting the unique effector functions of other isotypes for cancer immunotherapy, namely IgE and IgA mAbs.

The present review surveys approved antibody treatments and those under investigation, with an emphasis on antibodies featuring Fc mutations or modifications. Recent preclinical efforts to optimize antibody therapies through Fc mutations are also discussed. The potential use of IgE- and IgA-based therapeutics as effective cancer treatments as well as other novel approaches for optimization of Fc engagement is also discussed.

## Fc-Engineering

**Figure 1:**
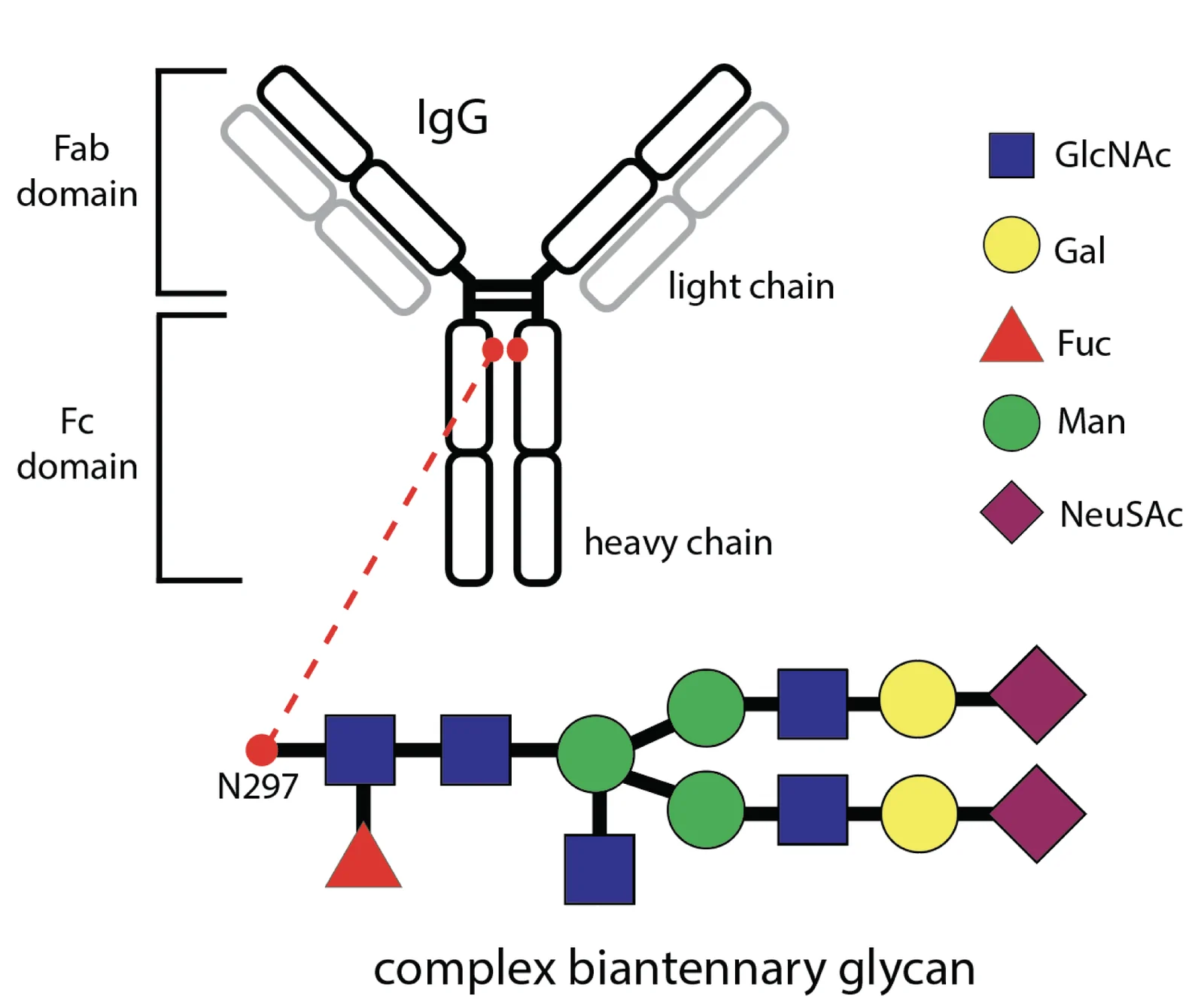
Complex biantennary glycan structure of IgG antibody N-acetylglucosamine (GlcNAc) is represented by the blue square. Galactose (Gal) is represented by the yellow circle. Fucose (Fuc) is represented by the red triangle. Mannose (Man) is represented by green circle. N-acetylneuraminic acid (Neu5Ac) is represented by the maroon diamond. This glycan is typically found at the asparagine 297 residue on the heavy chain.

**Figure 2:**
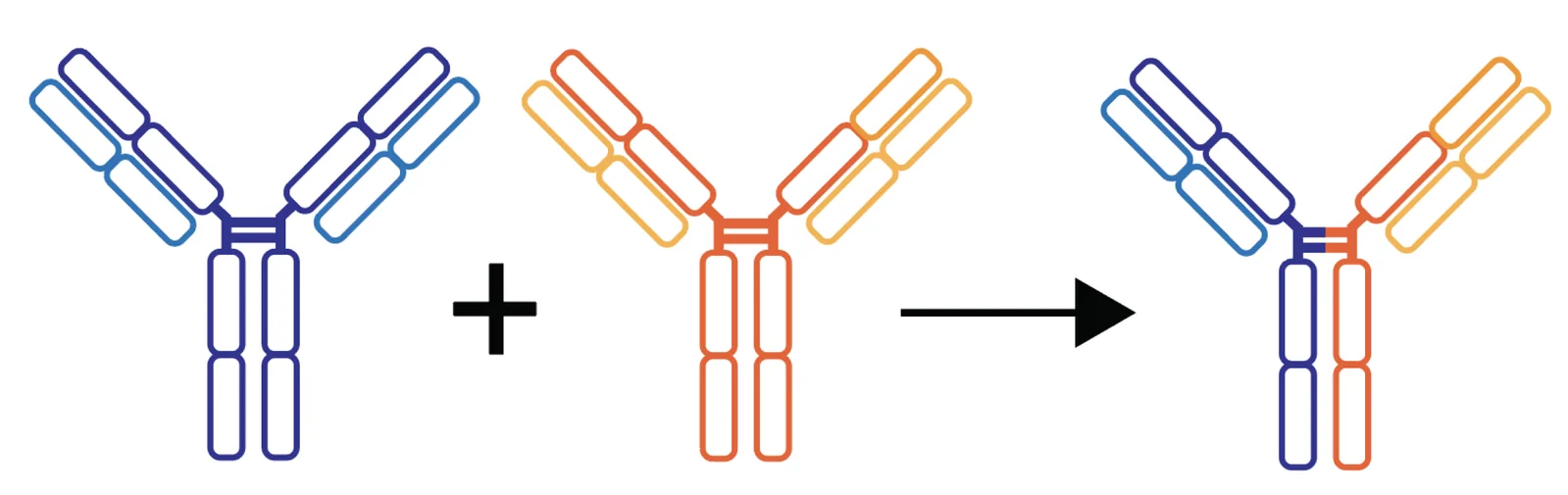
IgG4 arm-exchange The unique feature of IgG4 is that it can swap arms with another antibody, resulting in an antibody with two binding affinities with one antibody binding region per antigen affinity.

An antibody format is needed that is both clinically effective and commercially affordable. Depending on the potency of the antibody and its mechanism of action, amino acid substitutions in the Fc region of the IgG isotype have been introduced to enhance or silence Fc effector functions (Figure 1). Abrogating the Fc effector functions aims to reduce adverse side effects such as excessive collateral damage and off-target cytotoxicity caused by a strong inflammatory response triggered through Fc-related pathways. Other mutations have been introduced to increase the affinity of Ig for its cognate Fc receptor, thereby enhancing the killing of targeted cancer cells. Whether mutations are made to silence or enhance Fc-mediated effector functions largely depends on the target, as discussed below. Other amino acid substitutions have been introduced to improve the stability of antibody therapies. An important subset of these changes enables the generation of bispecific antibody constructs. Furthermore, the major glycosylation site within the CH2 domain of the Fc region, at asparagine residue 297 (Asn297), has been exploited to modulate Fc effector mechanisms [17]. The glycan installed on Asn297 is typically a complex biantennary glycan, often modified by fucosylation (Figure 2). When the glycosylation site Asn297 is mutated, eliminating N-linked glycosylation at that site, Fc effector functions are abolished [18]. On the other hand, when the core fucose is removed, binding to FcγRIIIa and ADCC are both improved [19]. Both Fc aglycosylation (removal of the N297 glycan) to silence Fc effector functions and Fc afucosylation (removal of core fucose) to enhance ADCC have been used to modify antibody-based cancer treatments.

## Mutations that attenuate Fc effector functions to reduce adverse side effects

Antibodies targeting immune checkpoints or self-antigens (overexpressed on cancer cells but also present on healthy cells) often require mutations that eliminate or drastically reduce Fc-mediated effector functions. However, the Fc region remains essential for extending the therapeutic's half-life. Several mutations have been used for this purpose. Commonly used amino acid substitutions, such as L234A, L235A, G237A, P329G, D265A, and P331S, are designed to disrupt FcγR and complement binding [20]. As described above, aglycosylation, achieved by mutating the N-linked glycosylation site at position N297 (e.g., N297A or N297G), is an effective strategy to attenuate Fc effector functions by eliminating the glycan moiety required for FcγR engagement.

Importantly, Fc attenuation is not a binary property. Commonly used substitutions such as LALA (L234A/L235A), LALA-PG (L234A/L235A/P329G), the L234F/L235E/P331S triple mutation, D265A, and removal of the N297 glycan (N297A/N297G) reduce FcγR and/or C1q engagement to differing degrees, but residual receptor binding and activity can persist [21]. P329G-containing variants such as LALA-PG are among the more complete, whereas LALA alone is comparatively leaky, and D265A can destabilize the Fc [22,23]. Aglycosylation through mutation of N297 markedly reduces low-affinity FcγR and C1q binding but does not necessarily eliminate all Fc-mediated activity: high-affinity FcγRI binding can persist, aglycosylated IgG1 can productively engage activating FcγRs, and immune complexes can still engage Fcγ receptors [24].

Several antibodies targeting the PD-L1-PD-1 axis, an important immune checkpoint, incorporate these effector-silencing mutations. Atezolizumab (anti-PD-L1), approved in the U.S. in 2016 for bladder cancer, uses an N297A mutation to suppress glycosylation at that site [20,25]. Durvalumab, a human IgG1 mAB that targets PD-L1, incorporates L234F, L235E, and P331S to reduce ADCC and CDC and was also approved for bladder cancer in 2017 [26–28]. Tislelizumab includes a combination of E233P, F234V, L235A, and D265A mutations to eliminate FcγR binding [29]. This mAB received U.S. approval in 2024 for esophageal squamous cell carcinoma [30]. Benmelstobart, another PD-L1-targeting antibody approved in China in 2024, uses D265A to reduce Fc function [31]. Other similarly modified antibodies include penpulimab (anti-PD-1) and tagitanlimab (anti-PD-L1), which harbor L234A, L235A, and G237A mutations and are proposed as therapies for nasopharyngeal carcinoma [29]. The choice to attenuate Fc function depends on the biology of the target. For PD-1 and PD-L1 blockade, the aim is to interrupt inhibitory signaling without depleting the effector T cells, antigen-presenting cells, and other immune populations that express the target and are needed for antitumor immunity; this is why most anti-PD-L1 antibodies use IgG4/S229P backbones and most anti-PD-L1 antibodies (e.g., atezolizumab and durvalumab) carry effector-reducing Fc modifications [32]. In contrast, anti-CTLA-4 antibodies can act not only by blocking inhibitory signaling but also through FcγR-dependent depletion of intratumoral regulatory T cells (which highly express CTLA-4) and through FcγR-driven myeloid remodeling [33,34]. The anti-CTLA-4 monoclonal ipilimumab has been associated with the high-affinity FcγRIIIa-V158F allele [35]. Fc silencing is therefore beneficial in some checkpoint contexts but may reduce antitumor activity in others.

Several bispecific antibodies have been designed with mutations to silence effector functions. Teclistamab, which targets both the B-cell maturation antigen (BCMA) and CD3 (co-receptor of the T-cell receptor) and was approved for multiple myeloma in 2022, has the following Fc modifications: L234A and L235A [36,37]. Similarly, mosunetuzumab, which targets CD20 (a B-cell antigen) and CD3 and was approved in the same year for follicular lymphoma, carries the N297G mutation to eliminate glycosylation at that site [38]. Odronextamab, another CD20×CD3 bispecific antibody with Fc-silencing modifications (E233P, F234V, L235A, and G236del), is currently under review in the U.S.A. [38]. Glofitamab, a bispecific antibody, has a unique format of Fab-Fc × Fab-Fab-Fc [39,40]. This bispecific antibody targets CD20 with high avidity, using two Fab regions for CD20 and one Fab for CD3. It was approved in 2023 for diffuse large B-cell lymphoma and has three Fc-silencing mutations: L234A, L235A, and P329G [29]. Additional bispecific antibodies engineered to reduce Fc effector functionality include the CD20xCD3 bispecific epcoritamab (L234F, L235E, D265A) and the GPRC5D (expressed by some myeloma cells) × CD3 bispecific talquetamab (F234A and L235A) [38].

## Mutations for stabilization of various antibody formats

**Figure 3:**
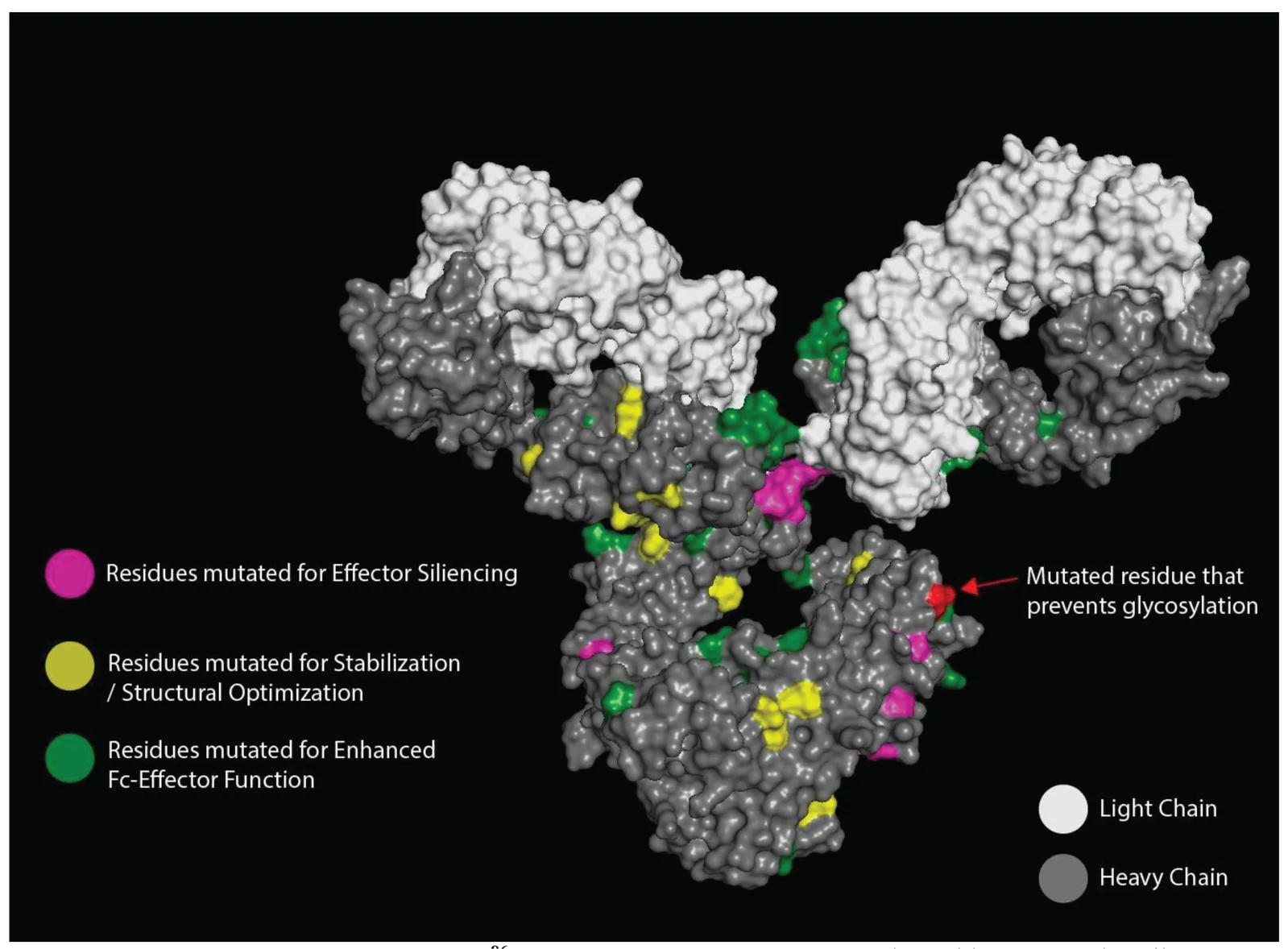
Monoclonal IgG antibody 1IGY [86] **with mutated residues labeled** The residues mutated to silence effector functions are labeled in magenta. The N297A mutation that removes glycosylation is highlighted in red. The residues mutated to enhance the stability and structure of antibodies are labeled in yellow. The residues that are mutated to enhance Fc-effector functions are colored green. The light chain is colored white to differentiate it from the heavy chain.

Stabilization of antibody structure is essential for therapeutic efficacy, especially for IgG4 antibodies. IgG4 is the least abundant IgG subclass in human blood and is prone to Fab-arm exchange in bispecific antibodies (Figure 3). Therefore, IgG4-based antibodies require precise heavy–heavy and heavy–light chain pairing. Specific mutations are introduced to enhance hinge stability, extend half-life, promote correct heterodimerization, and prevent chain mispairing.

A common mutation in IgG4-based mAbs is the replacement of the serine residue at position 228 in the hinge that connects the Fab and Fc regions with a proline to prevent Fab-arm exchange [41]. The IgG4 subtype is unique in that it can swap one half with another IgG4 molecule, becoming a monovalent bispecific antibody [41]. This is undesirable in a therapeutic context, where specificity and strong binding affinity to the antigen of choice are required for therapeutic success.

Pucotenlimab, an anti-PD-1 antibody approved in China in 2022, combines S228P with S254T, V308P, and N434A to not only stabilize the hinge but also extend half-life [42]. A treatment consisting of a mixture of two mAbs, iparomlimab and tuvonralimab, provides a combined PD-1/CTLA-4 blockade strategy [43]. In addition to the S228P mutation on iparomlimab (IgG4), mutations in the heavy chain of tuvonralimab (K147D, F170C, V173C, C220G, D399R, and K409E) and light chains (S131K, Q160C, S162C,and C214S) promote correct heavy- and light-chain pairing of the IgG1 antibody [38]. A further heavy-chain substitution, R255K, reduces FcRn binding to deliberately shorten the half-life of the anti-CTLA-4 component [44].

Heterodimeric Fc engineering is also critical in bispecific formats. Bispecific antibodies must solve two related assembly challenges: correct pairing of two different heavy chains and correct pairing of each heavy chain with its cognate light chain. Heterodimerization strategies address these in different ways [45]. Knobs-into-hole introduce steric complementarity into the CH3 interface. Glofitamab and mosunetuzumab use the knob-into-hole strategy, combining T366W on one heavy chain with T366S-L368A-Y407V on the other to ensure proper chain pairing and the formation of a stable heterodimer [38,46,47]. Electrostatic steering uses complementary charged substitutions [46]. Controlled Fab-arm exchange (the DuoBody approach) uses matched CH3 mutations (F405L on one chain, K409R on the other) to assemble a bispecific IgG from two parental antibodies under controlled redox conditions [46]. Both teclistamab and talquetamab use such CH3 mutations to promote heterodimer formation between the two different heavy chains [38,48,49]. CrossMab enforces correct light-chain pairing through domain crossover [46]. Common-light-chain formats remove one source of light-chain diversity. For example, Zenocutuzumab, a HER2xHER3 bispecific, has one light chain paired with two different heavy chains. The Fc of Zenocutuzumab has also been engineered to have two mutations, L351K and T366K, to promote heterodimerization [38,50]. Alternative CH3-interface scaffolds (e.g., SEED and BEAT) provide further options [46]. Elranatamab, a BCMA×CD3 bispecific, uses a glutathione-mediated Fab-arm exchange strategy for controlled heterodimer formation. Three arginine (R) mutations are introduced into the CD3 IgG1 antibody at residues 221, 228, and K409. If the CD3 IgG2 antibody format is used instead, four arginine mutations are introduced at residues 223, 225, 228, and K409. The BCMA-targeting antibody in the IgG1 format has three glutamic acid (E) residues added (221, 228,and L368E) and four in the IgG2 format (223E, 225E, 228E, and L368E) [38,51]. These platforms are not interchangeable, differing in valency and geometry, in whether they also solve light-chain mispairing, in manufacturability, and in their compatibility with a given Fc-effector profile [46,52].

These stabilizing modifications are essential for ensuring consistency in manufacturing, functional activity, and the clinical success of current bispecific and IgG4-based antibodies.

## Mutations that enhance Fc-mediated effector functions

In contrast with many antibody therapies targeting immune checkpoints, antibodies directed at tumor antigens often benefit from enhanced Fc-mediated effector functions such as ADCC and ADCP. This is particularly relevant to hematologic malignancies and HER2-positive cancers, where immune cell recruitment is a key component of the therapeutic mechanism.

An established approach to boosting Fc effector function is the removal of the core fucose from the Fc glycan, a strategy that enhances binding affinity to the activating FcγRIIIa receptor on NK cells, thereby increasing ADCC activity [19]. Obinutuzumab, a CD20-targeting antibody approved in the U.S. in 2013 for chronic lymphocytic leukemia, is produced in Chinese hamster ovary cells, where a bisecting N-acetylglucosamine is incorporated into oligosaccharides. This prevents the addition of fucose to the N-linked glycan at Asn297 [53]. Obinutuzumab is best described as a glycoengineered type II anti-CD20 antibody: the reduced core fucose enhances FcγRIIIa engagement and thereby ADCC and ADCP, while its type II mode of CD20 binding drives strong direct, non-apoptotic cell death and induces comparatively less CDC [54–56]. Its activity therefore reflects both glycoengineering-enhanced effector recruitment and type II-mediated direct cell death rather than ADCC alone. Other afucosylated antibodies include belantamab (anti-BCMA) and mogamulizumab, an anti-CCR4 antibody (targeting a chemokine receptor found on T cells) that prevents T cells from migrating to the skin and causing inflammation and skin lesions in certain rare skin cancers [57–59]. Both antibodies are afucosylated, with the goal of maximizing ADCC against target cells.

Amivantamab, a bispecific antibody that targets a mutant version of the epidermal growth factor receptor (EGFR exon 20 insertion mutation) and the receptor tyrosine kinase cMET, incorporates reduced fucosylation to enhance FcγRIIIa binding [60]. Tafasitamab, an anti-CD19 antibody, features S239D and I332E mutations to enhance ADCC [61]. Margetuximab, approved in 2020 for HER2-positive metastatic breast cancer, introduces F243L, R292P, Y300L, L235V, and P396L to enhance binding to the activating FcγRIIIa receptor and reduce affinity for the inhibitory FcγRIIB receptor, thereby increasing immune cell activation [62].

## Emerging Fc-engineering strategies

As antibody-based treatments continue to advance, efforts are ongoing to optimize these therapies through modification of their Fc region.

Emerging Fc engineering is best viewed as a toolkit rather than a single preferred direction. First, half-life and tissue distribution are tuned through pH-dependent FcRn binding, as shown in established variants YTE (M252Y/S254T/T256E) and LS (M428L/N434S), and newer variants such as Q311R/M428L and the REW variant (Q311R/M428E/N434W) described below [63–65]. Second, effector function is enhanced through activating-FcγR-optimized variants (e.g., S239D/I332E, S239D/A330L/I332E, and GASDALIE/GAALIE Fc mutants) or through afucosylation [66,67]. Third, complement activity is enhanced through hexamerization-promoting mutations (E345R/E430G/S440Y; see the discussion on Hexabody, below) [68]. Fourth, effector attenuation continues to be refined toward designs that minimize residual FcγR/C1q engagement while preserving FcRn binding [69]. Because each modification alters a receptor-binding, safety, and developability profile, the optimal Fc design is target- and indication-specific.

Two Fc mutations, Q311R and M428L, enhance the pH-dependent binding of human FcRn and extend the antibody’s circulatory half-life [70]. These mutations were tested in model therapeutic IgG antibodies (trastuzumab and aflibercept). These mutations also increase CDC and ADCC activity. For antibodies where Fc silencing is desirable, these mutations, in combination with established Fc-silencing mutations, retain enhanced pH-dependent binding to FcRn.

In the REW variant, the leucine at position 428 is instead replaced by glutamate (M428E) and a third substitution, N434W, is added [71]. The combination of these three amino acid substitutions improved circulatory half-life, enhanced targeting of mucosal sites, and increased complement-mediated killing of cancer cells. The Fc mutations enable needle-free delivery of the mutant IgG across respiratory epithelial barriers in FcRn transgenic mice.

## IgE as a novel Fc modality

Currently, there is growing interest in the clinical translation of IgE-based antibodies in cancer immunotherapy. With an Fc region distinct from that of IgG, IgE binds with high affinity to FcεRI receptors expressed on monocytes, macrophages, mast cells, basophils, and dendritic cells. Compared with the affinity of IgG for its respective Fc receptors, IgE binds to FcεRI receptors with 100–10,000-fold greater affinity. IgE-based antibody treatments could enable a more potent attack on cancer cells at lower doses, with higher-affinity interactions and the ability to access a different arsenal of immune cells.

The first IgE-based cancer antibody treatment directed against tumors expressing the folate receptor alpha antigen, MOV18, successfully completed its phase 1 clinical trial. It demonstrated an acceptable safety profile and showed preliminary antitumor activity [72]. These findings may provide momentum to further investigate the potential of IgE in treating other cancers.

Nevertheless, challenges remain in the implementation of IgE-based cancer treatments. These include a significantly reduced half-life and the potential to elicit type 1 hypersensitivity reactions. Unlike IgG, IgE lacks an inhibitory Fc receptor, which may contribute to a suboptimal safety profile.

## IgA as a novel Fc modality

There also is an interest in the use of IgA, which uniquely engages FcαRI (CD89) and recruits neutrophils. IgA has been used preclinically to treat neuroblastoma by targeting GD2, a ganglioside expressed on neuroblastoma cells [73]. A clinically approved treatment is an IgG1 anti-GD2 antibody; however, its use is associated with severe neuropathic pain due to binding GD2 on sensory neurons. Conversion of the antibody to an IgA isotype eliminated the neuropathic pain while maintaining strong neutrophil-mediated cytotoxicity. Introduction of specific mutations in the IgA molecule (N452G, P124R, C92S, N120T, I121L, and T122S), along with deletion of the tail piece of the CH3 domain (P131-Y148), increased the antibody's half-life, conferred greater thermal stability, and improved manufacturability, while still demonstrating *in vivo* efficacy by delaying or halting tumor growth.

IgA has also shown preclinical success in the treatment of pancreatic ductal adenocarcinoma (PDAC). Monoclonal IgA antibodies targeting epithelial cell adhesion molecule, a tumor-associated antigen expressed in PDAC patient samples, combined with CD47 blockade, a myeloid immune checkpoint, resulted in the activation of neutrophils and enhanced clearance of tumor cells [74].

## Possible areas of innovation

Several innovative preclinical strategies aim to optimize engagement of specific Fc receptors and extend serum half-life.

The biotechnology company Genmab developed an antibody platform that recapitulates the high-avidity, complement-activating geometry of naturally multimeric IgM antibodies in an IgG format. With the E345R mutation and two additional enhancing mutations, E430G and S440Y, these IgGs were shown to readily form hexamers upon encountering antigen on the cell surface, thereby greatly enhancing antibody-dependent CDC [75]. This antibody design platform is known as Hexabody [76]. This strategy has demonstrated potent antitumor activity in various cancers, including multiple myeloma [77–79]. However, a clinical trial using a Hexabody targeting two distinct epitopes on DR5, a death receptor that mediates apoptosis, was terminated due to safety concerns [80]. Nonetheless, this approach may still have potential for targets other than DR5.

One approach involves conjugating an antibody or antibody fragment specific for a target of interest to a nanobody (variable region derived from an alpaca heavy chain only antibody) specific for the light chain of immunoglobulins. We developed bispecific conjugates incorporating a nanobody specific for the mouse kappa light chain to recruit endogenous immunoglobulins from the circulation [81,82]. The recruited immunoglobulins can be of any specificity, encompass all isotypes, and elicit Fc-mediated effector functions, including ADCC, antibody-dependent cellular phagocytosis, and CDC. When applied in a mouse tumor model for checkpoint blockade (conjugation of VHH_kappa_ to a PD-L1- or CTLA4-specific nanobody), this treatment improved survival in mice inoculated with MC38 (colon carcinoma) and B16-F10 (melanoma) tumors compared with benchmark mAbs [83].

Another preclinical study introduced a novel class of engineered antibodies called IgE/G, which combines functional elements of both IgE and IgG1 isotypes to enhance antitumor immune responses [84]. Using HER2-specific antibody constructs, IgE/G antibodies retained the antigen-binding specificity of their parent antibodies while engaging both Fcγ receptors and FcεRI. *In vitro*, IgE/G antibodies mediated ADCC and degranulation, and *in vivo*, they inhibited tumor growth in humanized mouse models of HER2-positive cancer. Importantly, IgE/G antibodies exhibited favorable biophysical properties, manufacturability, and did not induce basophil activation in human blood samples, suggesting a low risk of acute allergic reactions. These findings highlight IgE/G as a promising new antibody format that may provide enhanced therapeutic efficacy by leveraging the complementary immune pathways driven by IgG and IgE.

## Conclusion

The evolution of antibody-based therapies is closely intertwined with Fc region engineering to refine immune engagement, therapeutic potency, and safety. While IgG remains the isotype of choice for most approved therapeutics owing to its long half-life and robust Fcγ receptor-mediated effector functions, next-generation Fc-engineering strategies continue to be at the forefront (Table 2). These strategies include Fc mutations fine-tuned to enhance or silence effector functions, glycoengineering, and structural modifications designed to improve stability and manufacturability, particularly in complex bispecific formats.

**Table 2:** Summary of Fc mutations and modifications in antibody therapeutics and their functional purposes

Category/purpose	Mutation(s)	Example antibody/format	Functional effect/purpose
Effector function silencing (reduce ADCC/CDC/ADCP)	L234A, L235A (‘LALA’)	Penpulimab, tagitanlimab	Disrupts FcγR and C1q binding; silences ADCC/CDC
	L234A, L235A, G237A	Penpulimab, tagitanlimab	Further reduces FcγR affinity for complete silencing
	L234F, L235E, P331S	Durvalumab	Minimizes FcγR and complement binding while retaining FcRn
	E233P, F234V, L235A, D265A	Tislelizumab	Eliminates FcγR engagement in checkpoint inhibitors
	D265A	Benmelstobart	Reduces FcγR and C1q binding
	N297A, N297G	Atezolizumab, mosunetuzumab	Removes N-linked glycosylation at N297 and markedly reduces FcγR/C1q engagement
	L234A, L235A, P329G	Glofitamab	Silences FcγR/C1q binding; prevents off-target activation
	F234A, L235A	Teclistamab	Prevents nonspecific T-cell activation (bispecific)
	E233P, F234V, L235A, G236del	Odronextamab	Eliminates FcγR binding in bispecific formats
	L234F, L235E, D265A	Epocritamab	Silences ADCC and complement activation
	F234A, L235A	Talquetamab	Effector-silenced Fc for bispecific targeting
Stabilization/structural optimization	S228P (IgG4 hinge mutation)	IgG4 mAbs (e.g., pucotenlimab and Iparomlimab)	Prevents Fab-arm exchange in IgG4 antibodies
	S254T, V308P, N434A	Pucotenlimab	Enhances hinge stability and prolongs half-life
	K147D, F170C, V173C, C220G, R255K, D399R, K409E (heavy chain)	Tuvonralimab	Promotes correct heavy-light chain pairing (IgG1)
	S131K, Q160C, S162C, C214S (light chain)	Tuvonralimab	Ensures proper light-chain pairing
	F405L, R409K	Teclistamab, talquetamab	Promotes heavy-chain heterodimerization
	T366W / T366S, L368A, Y407V (‘Knob-into-hole’)	Glofitamab, mosunetuzumab	Enables correct heterodimer formation
	L351K, T366K	Zenocutuzumab	Stabilizes HER2xHER3 bispecific heterodimer
	R221, R228, K409 (IgG1 CD3 arm); E221, E228, L368E (IgG1 BCMA arm)	Elranatamab	Enables glutathione-mediated Fab-arm pairing
Enhanced Fc-effector function (boost ADCC/ADCP/CDC)	Afucosylation (removal of core fucose from the Asn297 glycan)	Obinutuzumab, belantamab, mogamulizumab	Increases FcγRIIIa binding → enhanced ADCC
	S239D, I332E	Tafasitamab	Enhances FcγR binding and cytotoxicity
	F243L, R292P, Y300L, L235V, P396L	Margetuximab	Increases activating FcγRIIIa binding, decreases inhibitory FcγRIIB
	Q311R, M428L	Engineered trastuzumab, aflibercept	Enhances pH-dependent FcRn binding; increases half-life, ADCC & CDC
	Q311R, M428E, N434W	Further engineered trastuzumab, aflibercept	Further extends half-life, enhances mucosal targeting & CDC
Isotype-specific engineering (non-IgG)	N452G, P124R, C92S, N120T, I121L, T122S, Δ(P131-Y148)	IgA1 anti-GD2	Improves stability, manufacturability, half-life; reduces neuropathic pain
	-	MOV18 IgE	High-affinity FcεRI binding; recruits macrophages and mast cells
	- (IgEG hybrid Fc)	HER2-IgEG	Combines FcγR and FcεRI pathways for dual effector engagement
Novel/preclinical concepts	Fc-VHHκ nanobody conjugate	PD-L1 or CTLA-4 nanobody fusions	Recruits endogenous IgG for enhanced ADCC/ADCP/CDC
	IgEG (IgE/IgG1 chimera)	HER2-IgEG	Dual Fc receptor engagement; enhanced tumor killing and manufacturability

Expanding beyond the IgG paradigm involves exploring other isotypes, such as IgA and IgE, providing novel means of harnessing underutilized immune pathways, such as neutrophil-mediated cytotoxicity and potent FcεRI-driven immune activation. Preclinical and early clinical data, such as MOV18 IgE or IgA anti-GD2, demonstrate both the promise and complexity of these approaches. For IgA- and IgE-based therapeutics, challenges related to safety, half-life, and Fc receptor biology remain to be addressed. Hybrid antibody formats like IgE/G further exemplify how a strategic combination of isotype functionalities can create innovative therapeutic platforms that transcend traditional antibody classes.

Fc-engineering has thus become a central pillar in antibody drug development, moving beyond simple target binding to active modulation of the immune response. A diversified and highly engineered Fc toolbox will be a valuable asset in immunotherapy.

## Perspectives

- mAbs have transformed the landscape of cancer treatment, with all approved agents classified within the IgG subclass. These immunoglobulins exert antitumor activity primarily by interacting with Fcγ receptors present on immune effector cells.
- The present article presents a concise summary of the current landscape of Fc-engineered antibodies applied in cancer therapy. It reviews the locations of these mutations and their influence on Fc-mediated effector functions, including their activation and silencing. Additionally, the article provides an overview of clinically approved Fc-engineered antibodies utilized in cancer treatment.
- Advances include next-generation Fc variants for better effector control, novel glycan engineering, and alternative immunoglobulin isotypes (such as IgA) for improved stability and modulation of the immune response. Bringing these innovations into clinical use with safety checks will expand the applications of Fc-engineered antibodies in cancer treatment.

## Open Access

Open access for this article is provided via our Subscribe to Open programme, made possible through the support of our subscribing institutions. Learn more here: https://portlandpress.com/pages/open_access_options_and_prices#conditional.

## Abbreviations

ADCC: antibody-dependent cellular cytotoxicity
ADCP: antibody-dependent cellular phagocytosis
BCMA: B-cell maturation antigen
CDC: complement-dependent cytotoxicity
CRS: cytokine release syndrome
EGFR: epidermal growth factor receptor
EpCAM: epithelial cell adhesion molecule
Fc: fragment crystallizable
FcγR: Fc gamma receptors
FcRn: neonatal Fc receptor
Fuc: Fucose
Gal: Galactose
GlcNAc: N-acetylglucosamine
Ig: immunoglobulin
mAbs: monoclonal antibodies
Man: mannose
Neu5Ac: N-acetylneuraminic acid
PDAC: pancreatic ductal adenocarcinoma
